# Retraction Note: MicroRNA-27a-3p Modulates the Wnt/β-Catenin Signaling Pathway to Promote Epithelial-Mesenchymal Transition in Oral Squamous Carcinoma Stem Cells by Targeting SFRP1

**DOI:** 10.1038/s41598-020-77203-x

**Published:** 2020-12-01

**Authors:** Bin Qiao, Bao-Xia He, Jing-Hua Cai, Qian Tao, Alfred King-yin Lam

**Affiliations:** 1 Department of Stomatology, The First Affiliated Hospital of Zhengzhou University, Zhengzhou, 450052 P. R. China; 2 Department of Oral and Maxillofacial Surgery, Guanghua School and Hospital of Stomatology; Guangdong Provincial Key Laboratory of Oral Diseases, Sun Yat-Sen University, Guangzhou, 510055 P. R. China; 3 Department of Pharmacy, Henan Cancer Hospital/Affiliated Cancer Hospital of Zhengzhou University, Zhengzhou, 450052 P.R. China; 4 Cancer Molecular Pathology, School of Medicine and Menzies Health Institute Queensland, Griffith University, Gold Coast, 4222 Australia

Retraction of: *Scientific Reports* 10.1038/srep44688, published online 20 April 2017

The authors have retracted this Article.

After publication they became aware that the oral cancer cell line had been contaminated with HeLa cells. In addition, there are irregularities in a number of figures, specifically:

- Apparent overlap of the flow cytometry plots shown in Figure 1A and 1B which are for different cell lines
- Duplication of blots between Figure 4A and Figure 4C
- Duplication of blots between Figure 5B and Figure 5D
- Duplication of blots between Figure 7B and Figure 7D
- Duplication of parts of panels both within Figures 8 and 9 and between Figures 8 and 9 and parts of panels in figures in other articles^1–4^

All authors agree to this retraction
